# Oxidative stress increases the risk of pancreatic *β* cell damage in chronic renal hypertensive rats

**DOI:** 10.14814/phy2.12900

**Published:** 2016-08-17

**Authors:** Shan Gao, Byung M. Park, Seung A. Cha, Ui J. Bae, Byung H. Park, Woo H. Park, Suhn H. Kim

**Affiliations:** ^1^ Department of PhysiologyChonbuk National University Medical SchoolJeonjuKorea; ^2^ Department of PharmacologyTaishan Medical UniversityShandongChina; ^3^ Department of BiochemistryResearch Institute for Endocrine SciencesChonbuk National University Medical SchoolJeonjuKorea

**Keywords:** 8‐hydroxydeoxyguanosine, advanced glycation end products, angiotensin II, diabetes, H_2_O_2_, hypertension, NADPH oxidase‐4, oxidative stress, *β* cell

## Abstract

Hypertension often occurs in conjunction with insulin resistance. The purpose of this study was to evaluate whether sustained renal hypertension increases the risk of diabetes mellitus in rats, and to define the underlying mechanisms. Two‐kidney, one‐clip hypertensive (2K1C) rats received captopril (50 mg/kg/day), *α*‐lipoic acid (100 mg/kg/day), or vehicle treatment for 3 months after surgery. Blood pressure was measured by tail cuff plethysmography. Oral glucose tolerance test (OGTT), immunohistochemistry, and western blotting were performed. In addition, insulin secretion from islet cells was measured. OGTT yielded abnormal results, and the number of islet cells and the size of pancreatic *β*/*α* cells were decreased in 2K1C rats. Basal insulin levels were also reduced in the plasma. Insulin secretion from pancreatic islet cells in response to high glucose was also attenuated in 2K1C rats compared with sham rats. The levels of oxidative stress markers, including 8‐hydroxydeoxyguanosine and NADPH oxidase‐4, were increased in pancreatic tissue and pancreatic islets in 2K1C rats. The abnormalities observed in 2K1C rats were improved by captopril or *α*‐lipoic acid treatment. These findings indicate that sustained renal hypertension may lead to pancreatic dysfunction, increasing oxidative stress in pancreatic islets.

## Introduction

Hypertension and cardiovascular diseases are the leading causes of morbidity and mortality (Kannel and Wilson 1995). Hypertension often occurs in conjunction with insulin resistance (Carlsson et al. 1998) and other components of the cardiometabolic syndrome (Cersosimo and DeFronzo 2006). Angiotensin II (Ang II) is the major peptide hormone of the renin–angiotensin system (RAS) and plays a pivotal role in the pathogenesis of hypertension and insulin resistance (Richey et al. 1999; Ran et al. 2006). Studies have indicated that Ang II inhibits insulin activity in vascular and skeletal muscle tissues, in part, by interfering with insulin signaling through the phosphatidylinositol 3‐kinase and protein kinase B pathway (Ogihara et al. 2002; Sowers 2004). Ang II also suppresses adiponectin production, which impairs insulin sensitivity (Ran et al. 2006). Insulin resistance promotes hypertension by up‐regulating vascular Ang II type 1 receptor (AT_1_R) (Nickenig et al. 1998). Most of the studies were performed in animals suffering with diabetic mellitus as a causal factor of hypertension. However, there is a few evidence that hypertension causes an impairment of insulin secretion leading to diabetes mellitus. Conen et al. (2007) reported hypertensive patients showing an approximately 3.3‐fold increased risk of new‐onset diabetes, relative to nonhypertensive patients. This study seems to be the first report showing hypertension to become a causal factor of diabetes mellitus.

It has been indicated by experimental evidence that oxidative stress plays an important role in the pathophysiology of hypertension and target organ damage in hypertension, especially focusing on atherosclerosis, heart disease, renal failure, and cerebrovascular disease (Dinh et al. 2014; Rubattu et al. 2015; Sinha and Dabla 2015). Oxidative stress induces modification of DNA, protein, and lipid peroxidation, which contribute to the pathogenesis of various diseases (Nickenig and Harrison 2002). Ang II signaling promotes the production of reactive oxygen species (ROS) via NADPH oxidase (NOX) in adipose tissue, skeletal muscle, cardiovascular tissue (Nickenig and Harrison 2002; Sowers 2002, 2004), and the pancreas (Chan and Leung 2011). Tikellis et al. reported that components of RAS are expressed in the pancreas and that the RAS blockade has beneficial effects on pancreatic structure and function (Tikellis et al. 2004). Moreover, Ang II suppresses glucose‐induced insulin secretion (Fliser et al. 1997) and reduces blood flow to islets (Carlsson et al. 1998). These findings suggest that oxidative stress induced by the Ang II signaling pathway is one of the causal factors connecting hypertension to the impairment of insulin secretion and/or production leading to diabetic condition. We hypothesized that hypertension‐induced oxidative stress may be a predisposing factor for diabetes. To test this hypothesis, oral glucose tolerance test (OGTT) was performed in two‐kidney one‐clip (2K1C) rats, 5 weeks, 3 months, and 6 months after surgery (postop 6‐month). The fasting blood glucose (FBS) level in 2K1C rats tended to be high without significance and the impairment of OGTT was more prominent in postop 6‐month 2K1C rats (Table 1). Therefore, we examined the morphology, number, and insulin secretion of islets in postop 6‐month 2K1C rats.

**Table 1:** Fasting blood sugar, area under curve during oral glucose tolerance test, plasma hormone levels in sham and two‐kidney one‐clip hypertensive rats

	Sham	5 weeks	3 months	6 months
FBS (mg/dL)	96.0 ± 8.3	112.3 ± 5.9	102.4 ± 5.9	104.6 ± 8.0
AUC of OGTT (mg/dL/3 h)	367.2 ± 20.5	385.4 ± 25.2	415.4 ± 25.6	435.7 ± 17.2*
P_Ang II_ (pg/mL)	43.9 ± 5.5	925.5 ± 65.6*	824.2 ± 219.3	56.0 ± 15.7
P_Aldo_ (pg/mL)	235.0 ± 70.5	1454.6 ± 165.6*	925.2 ± 89.3*	168.4 ± 74.9

Values are mean ± SEM of sham rats and two‐kidney one‐clip hypertensive rats (5 weeks, 3 months, and 6 months after surgery) (*n* = 4–8). FBS, fasting blood sugar; AUC of OGTT, area under the curve of oral glucose tolerance test; P_Ang II_, P_Aldo_, plasma levels of angiotensin II and aldosterone levels, respectively. *versus Sham rats, *P *<* *0.05.

## Methods

### Animals and treatments

Male Sprague–Dawley (SD) rats aged 5–6 weeks were purchased from Orient Bio (Seoungnam, Korea) and were housed in a temperature‐controlled room with a 12:12‐h light–dark cycle. The animals had free access to standard laboratory chow (5L79 Purina rat & mouse 18% chow; Charles River Laboratories, Wilmington, MA) and water. All experimental protocols conformed to the National Institutes of Health Guide for the Care and Use of Laboratory Animals (NIH publication No. 85–23, revised 1996) and were approved by Chonbuk National University Medical School.

Following an acclimatization period of 3 days, surgery was performed to make 2K1C hypertensive rats. Animals were anesthetized with a mixture of ketamine and xylazine (9:1, 2 mL/kg), then a silver clip (0.2 mm ID) was inserted on the left renal artery (Yuan et al. 2008). Sham rats received the same treatment except for placement of silver clips. Systolic blood pressure (SBP) was measured once a week by tail cuff plethysmography (Power Lab 2/20, AD instruments, Australia), and rats with SBP >140 mmHg were considered hypertensive and used for experiments. Three months after surgery, 2K1C rats were randomly divided into three groups: group 1 rats received vehicle (2K1C‐vehicle rats, *n* = 13), group 2 rats received captopril (Ang II‐converting enzyme inhibitor, 50 mg/kg/day, *n* = 10) (2K1C‐captopril rats), and group 3 rats received *α*‐lipoic acid (antioxidant, 100 mg/kg/day, *n* = 10) (Yu et al. 2012) (2K1C‐lipoic acid rats) orally for 3 months. Sham rats were maintained for 6 months after surgery as a control group (*n* = 7).

### OGTT and plasma hormone levels

A 50% glucose solution (2.0 g/kg) was orally administered to overnight‐fasted rats. Blood was sequentially collected from the tail vein before the administration of glucose and 0.5, 1, 2, and 3 h after glucose administration. Blood glucose levels were measured using a hand‐held glucometer (Accucheck, Roche Diagnostic, Basel, Switzerland). Levels of insulin, Ang II, and aldosterone were measured in the plasma using an insulin ELISA kit (Millipore, St. Charles, Missouri), Ang II ELISA kit (Enzo Life Sciences, Plymouth Meeting, PA), and aldosterone radioimmunoassay kit (DiaSorin, Northwestern Avenue Stillwater, MN), respectively.

### Glucose‐induced insulin secretion by islet cells in vitro

Pancreatic islets were isolated from sham and 2K1C rats as described previously (Kim et al. 2007). Rats were anesthetized with a mixture of ketamine and xylazine, then the common bile duct was cannulated. After cannulation, the bile duct was infused with 50 ml of collagenase solution (0.15 mg/mL collagenase P [Roche, Indianapolis, IN] in Hanks’ balanced salt solution supplemented with 1 g/L glucose and 0.2 g/L bovine serum albumin [BSA]). After infusion, the pancreas was removed and incubated in a water bath at 37°C for 30 min with gentle shaking every 10 min. The digested pancreas was washed with Tris‐HCl with Tween (TBST), and the islets were separated by centrifugation over a discontinuous Ficoll gradient. Isolated pancreatic islets were washed three times in Krebs–Ringer bicarbonate buffer (KRB) (25 mmol/L HEPES, 115 mmol/L NaCl, 24 mmol/L NaHCO_3_, 5 mmol/L KCl, 1 mmol/L MgCl_2_, 2.5 mmol/L CaCl_2_, 3 mmol/L D‐glucose, and 0.1% BSA, pH 7.4). Islets were incubated fivefold in 1.5 mL Eppendorf tube (10 islets/tube) in KRB buffer with 5.5 mmol/L or 20 mmol/L glucose at 37°C for 60 min. Finally, the islet samples were centrifuged and supernatant was collected for insulin assays.

### Immunohistochemistry of pancreatic islet cells

Pancreatic tissue was fixed in 10% formalin overnight and then embedded in paraffin. Ten 4‐μm sections were cut from each tissue block (*n* = 5 for each group). For immunohistochemistry, the tissue sections were blocked with peroxidase blocking agent for 5 min and washed with TBST, then blocked with protein blocking serum‐free buffer (DAKO, Carpinteria, CA) for 5 min. Sections were incubated in primary antibodies against insulin (Santa Cruz Biotechnology, Dallas, TX), glucagon (Bioworld, St. Louis Park, MN), NOX‐4 (Abcam, Cambridge, MA), or 8‐hydroxydeoxyguanosine (8‐OHdG; Abcam) for 2 h at room temperature. After washing with TBST, samples were then incubated in secondary antibodies (DAKO) at room temperature for 30 min, then the chromogen was added. All sections were H&E stained and observed at 200× magnification with a light microscope (Nikon, Tokyo, Japan). Islet areas and entire section areas were measured using iSolution DT 36 software (Carl Zeiss, Oberkochen, Germany) (Ka et al. 2015).

### Measurement of pancreatic oxidative stress markers

Rats were killed and pancreatic tissue was removed and stored at −80°C until use. To measure oxidative stress, pancreatic tissue was cut into small pieces, homogenized, and M‐PER lysed in T‐PER mammalian protein extraction reagent (Thermo Scientific, Rockford, IL) according to the manufacturer's instructions. Hydrogen peroxide (H_2_O_2_) and Mn‐superoxide dismutase (Mn‐SOD) were measured in pancreatic tissue using ELISA assay kits (Enzo Life Sciences). Advanced glycation end products (AGE), 8‐OHdG, and oxygen radical antioxidant capacity (ORAC) were measured using ELISA assay kits (Cell Biolabs, San Diego, CA).

For western blot analysis, pancreatic tissue was cut into small pieces, homogenized, and lysed using T‐PER mammalian protein extraction reagent (Thermo Scientific). After separation by gradient SDS‐polyacrylamide gel electrophoresis, proteins were transferred to an Immobilon‐polyvinylidene fluoride membrane and blocked with TBST containing 5% skim milk. The membranes were incubated with primary antibodies against AT_1_R, AT_2_R (Sigma‐Aldrich, St. Louis, MA), Mn‐SOD (Stressgen Biotechnologies, Victoria, BC), NOX‐4 (Santa Cruz Biotechnology), and GAPDH (Sigma‐Aldrich). Proteins were detected by incubation in horseradish peroxidase‐conjugated secondary antibodies (Zymed, South San Francisco, CA) at room temperature for 1 h. Immunoreactivity was detected using chemiluminescence and analyzed quantitatively using Image J (NIH, Bethesda, MD) (Shah et al. 2012).

### Statistical analysis

Results are presented as means ± SEM. Statistical significance of the differences was assessed using analysis of variance followed by the Bonferroni multiple comparison test. Student's *t* test was also used. The statistical significance was set at *P *<* *0.05.

## Results

### OGTT in 2K1C rats

**Figure 1:**
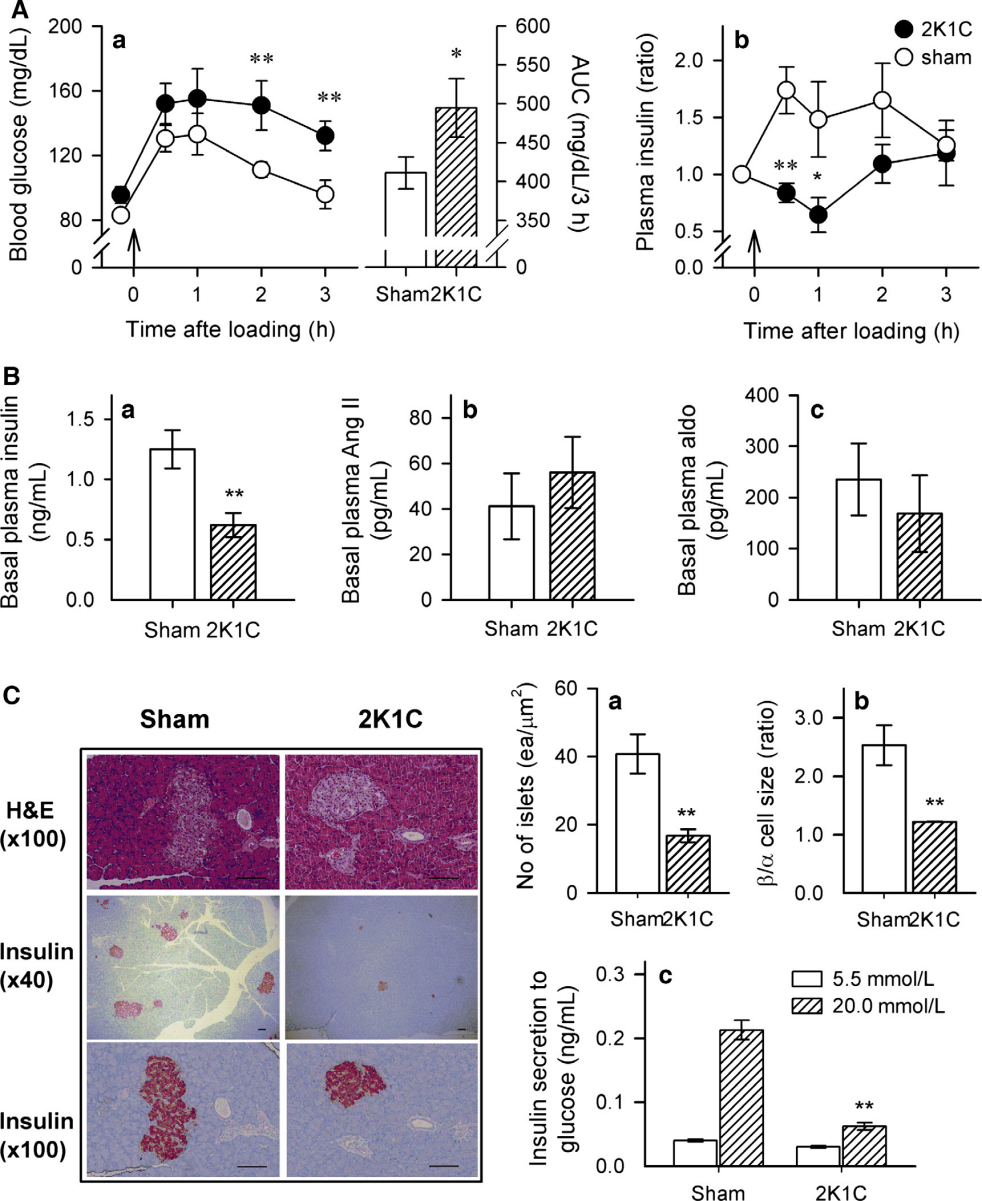
(A) Blood glucose level (left), area under the curve of glucose disposal (AUC, right) (a), and changes in plasma insulin level (b) during the glucose tolerance test (OGTT) in sham and 2K1C rats. (B) Comparison of basal plasma levels of insulin (a), angiotensin (Ang) II (b), and aldosterone (c) in both groups. (C) Comparison of the total number of islets labeled with an insulin antibody (a), ratio of the size of *β*/*α* cells (b), and glucose‐stimulated insulin secretion (c) in both groups. Pancreatic *α* and *β* cells were labeled with primary antibodies against glucagon and insulin, respectively. The number of islets was calculated as the islet number divided by the size of the pancreatic tissue. Insulin secretion was measured using isolated islets incubated with 5.5 or 20 mmol/L glucose at 37°C for 1 h. Data are presented as mean ± SEM of 7–9 rats in each group. 2K1C, two‐kidney, one‐clip hypertensive rats; sham, age‐matched sham rats. Bar indicates 100 μm. *versus sham rats, *P <* *0.05, **P <* *0.01.

During the first 2 months after surgery, there was no difference in body weight between sham and 2K1C rats (data not shown). After the third month, the body weight of 2K1C rats was significantly reduced compared with sham rats (429.7 ± 32.3 g vs. 492.7 ± 12.2 g, *P *<* *0.05). 2K1C rats with a SBP of >140 mmHg were allocated to the hypertensive group. The SBP of hypertensive rats gradually increased up to 190 mmHg and was maintained at that level throughout the experiment (190.0 ± 12.1 mmHg, *n* = 17 vs. 123.2 ± 0.8 mmHg, *n* = 9, *P *<* *0.01). The levels of glucose and insulin in response to glucose administration in postop 6‐month rats are shown in Figure 1. The blood glucose level after 8 h fasting (FBS) was not different between 2K1C and sham rats (Fig. 1Aa). The blood glucose levels were substantially increased in 2K1C and sham rats 30 min after glucose administration (Fig. 1Aa). The elevated glucose level of 2K1C rats was slightly reduced 3 h after glucose administration, while the elevated glucose level significantly decreased to basal levels in sham rats. Similarly, the glucose area under the curve (AUC) of 2KlC rats was higher than that of sham rats. Glucose administration increased the plasma insulin level of sham rats, but the insulin level of 2K1C rats did not significantly change (Fig. 1Ab). Basal plasma insulin levels were lower in 2K1C rats than sham rats (Fig. 1Ba). There were no significant differences in plasma Ang II (Fig. 1Bb) and aldosterone levels (Fig. 1Bc) between the two groups. In postop 5‐week and 3‐month 2K1C rats, the FBS levels tended to be high without significance and the impairment of OGTT was not prominent even though plasma levels of Ang II and aldosterone were higher than sham rats (Table 1). Therefore, we examined the morphology, number, and insulin secretion of islets in postop 6‐month 2K1C rats.

### Change in pancreatic islets in 2K1C rats

To evaluate the function of pancreatic *β* cells, the number of pancreatic islets and size of pancreatic *α* and *β* cells were determined using immunohistochemistry. As shown in Figure 1C, the number of Langerhans islets (Fig. 1Ca) and the size of *β*/*α* cells (Fig. 1Cb) were markedly decreased in 2K1C rats. In addition, glucose‐stimulated insulin secretion by *β* cells was also reduced in 2K1C rats (20 mmol/L) compared with sham rats (Fig. 1Cc).

### Effect of captopril or *α*‐lipoic acid treatment on SBP, glucose tolerance, and plasma hormone levels in 2K1C rats

**Figure 2:**
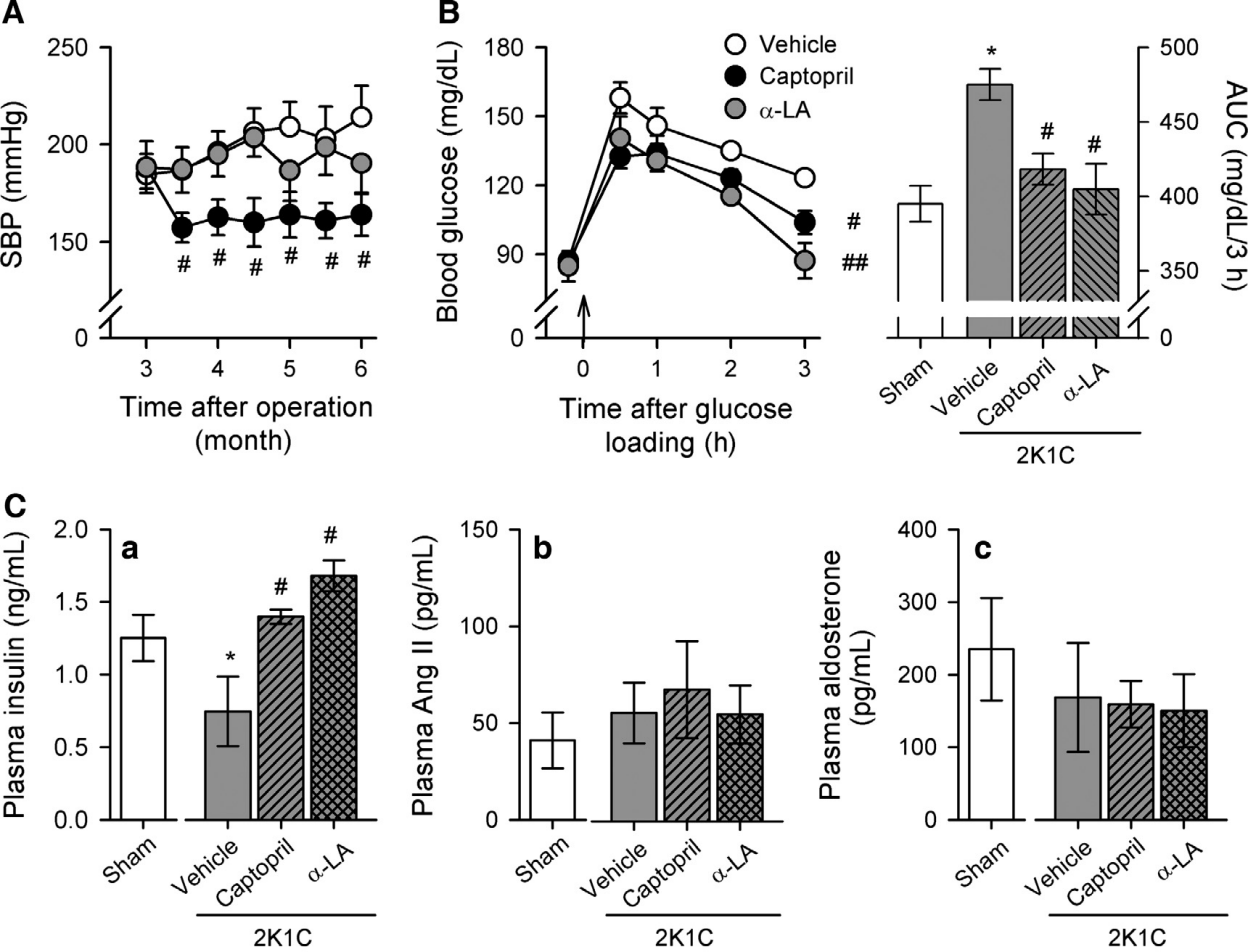
Changes in systolic blood pressure (SBP) (A) and blood glucose level (B) during OGTT in sham and 2K1C rats treated with vehicle, captopril, or *α*‐lipoic acid. (C) Basal plasma levels of insulin (a), Ang II (b), and aldosterone (c) among four groups. Data are presented as mean ± SEM of 7–10 rats in each group. *α*‐LA,* α*‐lipoic acid. *versus sham rats, *P *<* *0.05; ^#^versus 2K1C rats administered with vehicle, ^#^ *P *<* *0.05, ^##^ *P *<* *0.01.

To evaluate whether pancreatic *β*‐cell dysfunction is caused by hypertension or oxidative stress, postop 3‐month 2K1C rats received vehicle, captopril, or *α*‐lipoic acid treatment for 3 months. The SBP was lower in 2K1C‐captopril rats than 2KIC‐vehicle rats (Fig. 2A). Treatment with *α*‐lipoic acid did not have a significant effect on SBP (Fig. 2A). The basal blood glucose level was similar after 8 h fasting and the blood glucose level increased significantly after glucose administration in all groups. The peak of glucose increase after glucose administration was not different. However, blood glucose levels and the AUC during OGTT were significantly lower 3 h after glucose administration in 2K1C‐captopril rats and 2K1C‐lipoic acid rats compared with 2K1C‐vehicle rats (Fig. 2B). Basal insulin levels in the plasma were also higher in 2K1C‐captopril and 2K1C‐lipoic acid rats compared with 2K1C‐vehicle rats (Fig. 2Ca). However, there were no significant differences in plasma Ang II (Fig. 2Cb) and aldosterone levels (Fig. 2Cc) among the four groups.

### Morphological changes of islets in 2K1C rats after vehicle, captopril, or *α*‐lipoic acid treatment

**Figure 3:**
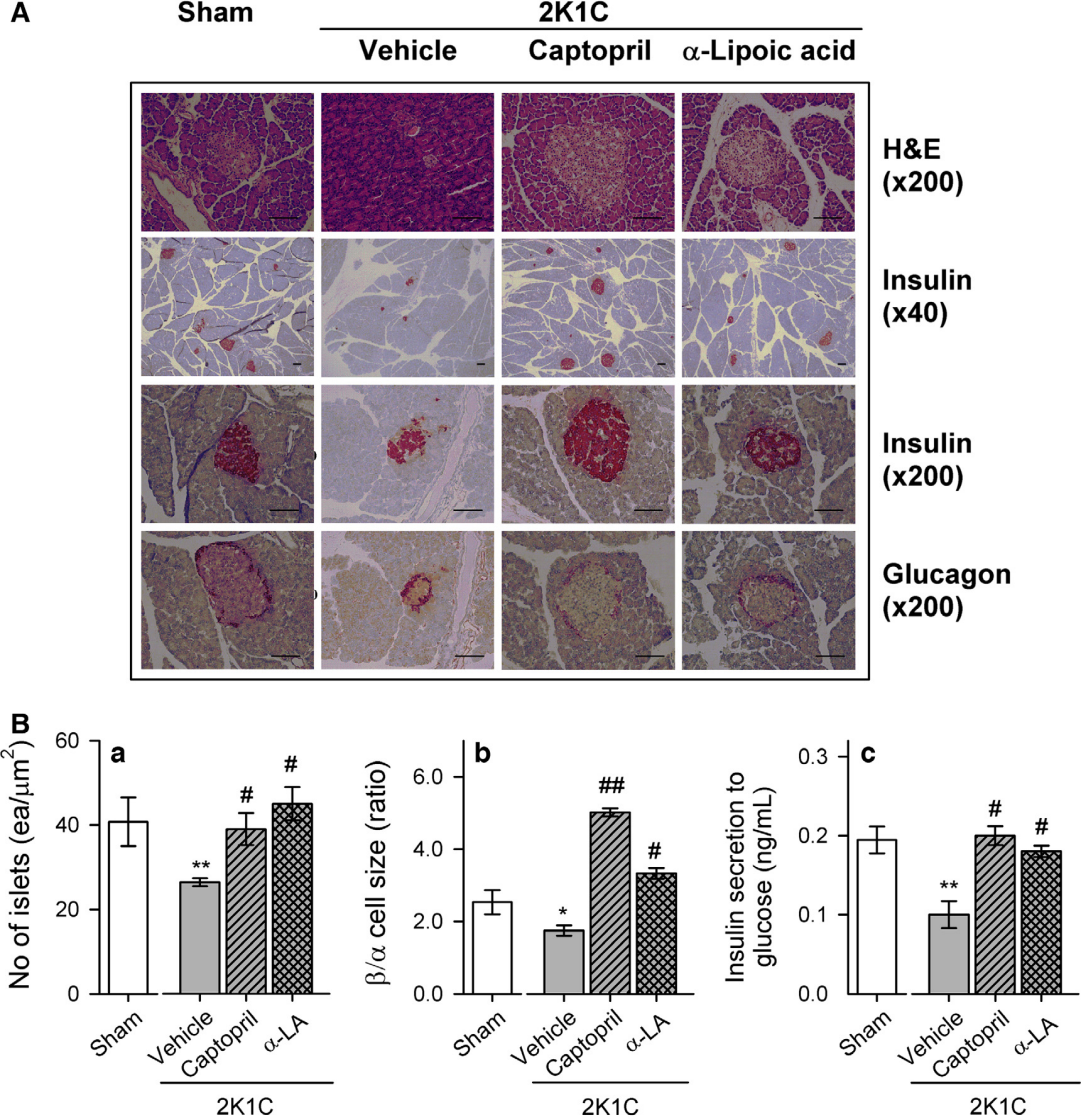
(A) Representative pancreatic islets stained with H&E and immunohistochemistry for insulin (*β* cell) and glucagon (*α* cell) from sham and 2K1C rats treated with vehicle, captopril, or *α*‐lipoic acid. (B) Quantification of the total number of islets (a), the ratio of *β*/*α*‐cell size (b), and high‐glucose‐induced insulin secretion (c) using iSolution DT 36 software. Values are expressed as mean ± SEM of seven rats in each group. Bar indicates 100 μm. *versus sham group, *P <* *0.05, **P <* *0.01; ^#^versus 2K1C rats fed vehicle, ^#^ *P *<* *0.05, ^##^ *P *<* *0.01.

Figure 3A shows the morphological changes in pancreatic islets of 2K1C rats after water, captopril, or *α*‐lipoic acid administration compared with sham rats. The number of pancreatic islets and the size of pancreatic *β*/*α* cells were decreased in 2K1C rats, and this was improved by captopril or *α*‐lipoic acid treatment (Fig. 3A). Quantification of pancreatic islets and pancreatic *β*/*α* cell size confirmed a significant improvement after treatment with captopril or *α*‐lipoic acid in 2K1C rats (Fig. 3Ba and Bb). Ex vivo*,* glucose‐stimulated insulin secretion (20 mmol/L) by isolated islet cells was improved to control levels in 2K1C‐captopril rats and 2K1C‐lipoic acid rats (Fig. 3Bc).

### Effect of vehicle, captopril, or *α*‐lipoic acid treatment on oxidative stress in the pancreas of 2K1C rats

**Figure 4:**
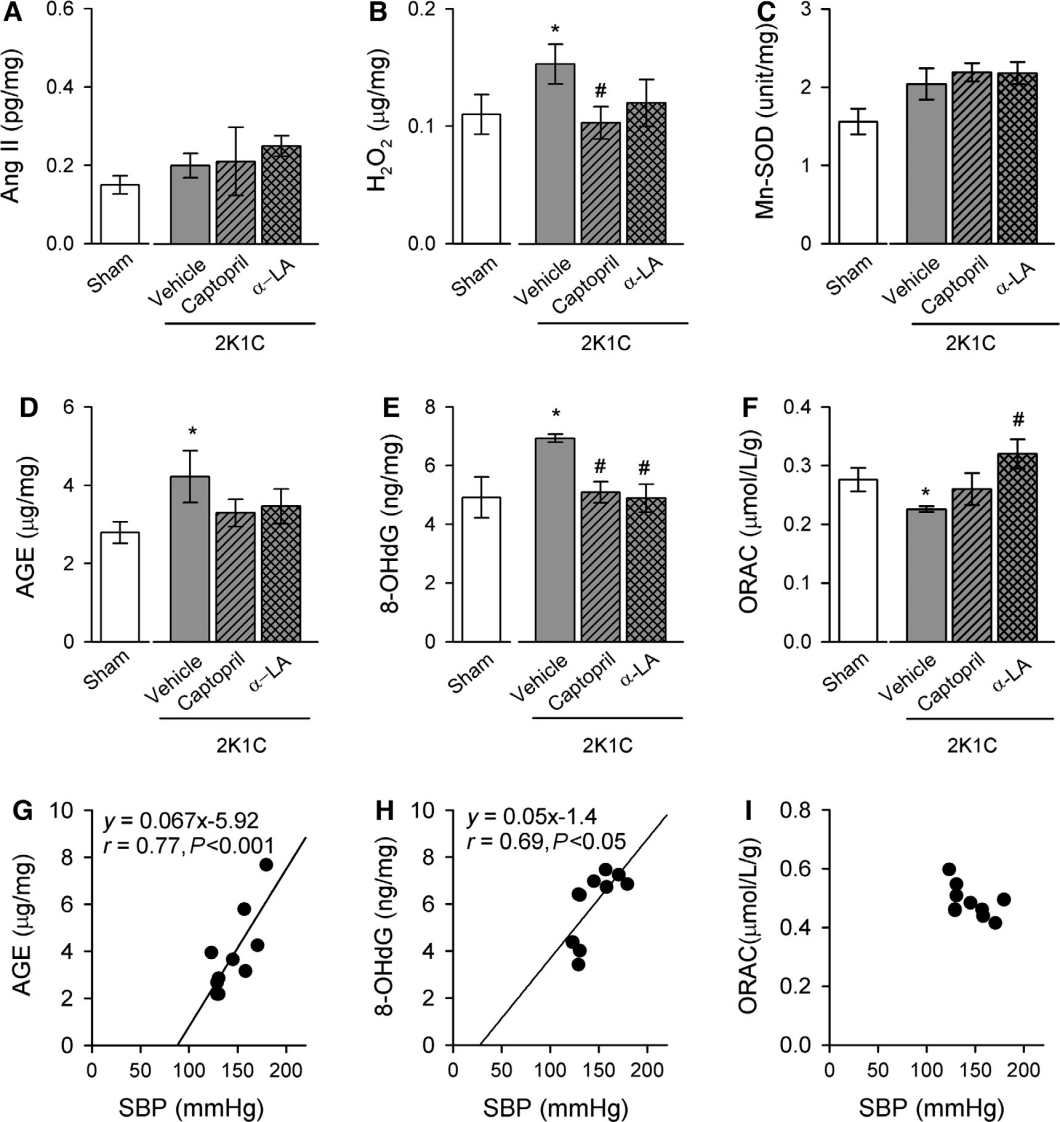
Changes in pancreatic levels of Ang II (A), hydrogen peroxide (B), Mn‐SOD (C), AGE (D), 8‐OHdG (E), and ORAC (F) of sham and 2K1C rats treated with water, captopril, or *α*‐lipoic acid. Correlation between SBP and AGE (G), 8‐OHdG (H), or ORAC level (I). Data are expressed as mean ± SEM of six rats in each group. Ang II, angiotensin II; Mn‐SOD, Mn‐superoxide dismutase; AGE, advanced glycation end product; 8‐OHdG, 8‐hydroxydeoxyguanosine; ORAC, oxygen radical antioxidant capacity. *versus sham group, *P *<* *0.05; ^#^versus 2K1C rats fed vehicle, *P *<* *0.05.

To evaluate oxidative stress in pancreatic tissues, Ang II, H_2_O_2_, Mn‐SOD, AGEs, 8‐OHdG, and ORAC levels were measured. The level of H_2_O_2_ in pancreatic tissues increased in 2K1C‐vehicle rats but not in 2K1C‐captopril and 2K1C‐lipoic acid rats (Fig. 4B). Ang II and Mn‐SOD levels did not differ significantly among the four groups (Fig. 4A). AGE formation is increased by hyperglycemia; therefore, AGEs are implicated in diabetes and pancreatic *β*‐cell dysfunction (Schleicher et al. 1997). The AGE level was significantly higher in pancreatic tissue of 2K1C rats compared with sham rats (Fig. 4D) and there was a positive relationship between AGE levels and SBP (*y* = 0.067*x* − 5.92, *r* = 0.77, *P *<* *0.001 for sham and 2K1C rats) (Fig. 4G). 8‐OHdG is a biomarker of oxidative DNA damage and was also higher in pancreatic tissue of 2K1C rats compared with sham rats (Fig. 4E). There was a positive correlation between 8‐OHdG levels and SBP (*y* = 0.05*x* − 1.14, *r* = 0.69, *P *<* *0.05) (Fig. 4H). The ORAC was lower in pancreatic tissue of 2K1C rats (Fig. 4F), and there was no significant correlation between ORAC levels and SBP (Fig. 4I). Captopril or *α*‐lipoic acid treatment reduced AGE and 8‐OHdG levels (Fig. 4D and E) and enhanced ORAC levels in the pancreas (Fig. 4F).

**Figure 5:**
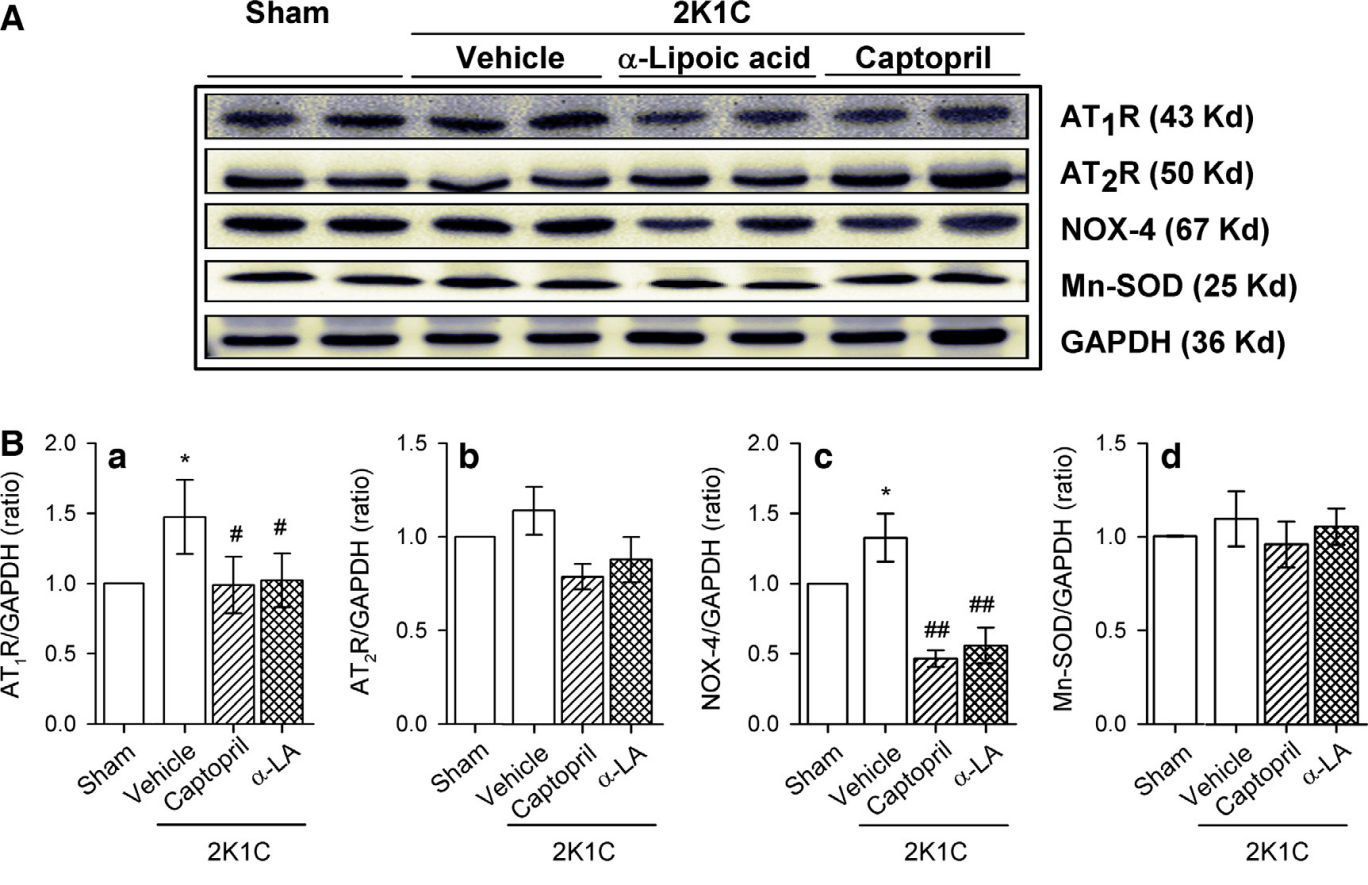
(A) Western blot showing AT_1_R, AT_2_R, NOX‐4, and Mn‐SOD protein expression in sham and 2K1C rats treated with vehicle, captopril, or *α*‐lipoic acid. (B) Quantitative analysis of western blotting using Image J. Values are expressed as mean ± SEM of 5–7 rats in each group. AT1R, AT2R, angiotensin II type 1 and type 2 receptor, respectively; NOX 4, NADPH oxidase 4. *versus sham group, *P *<* *0.05; ^#^versus 2K1C rats fed vehicle, ^#^ *P *<* *0.05, ^##^ *P *<* *0.01.

**Figure 6:**
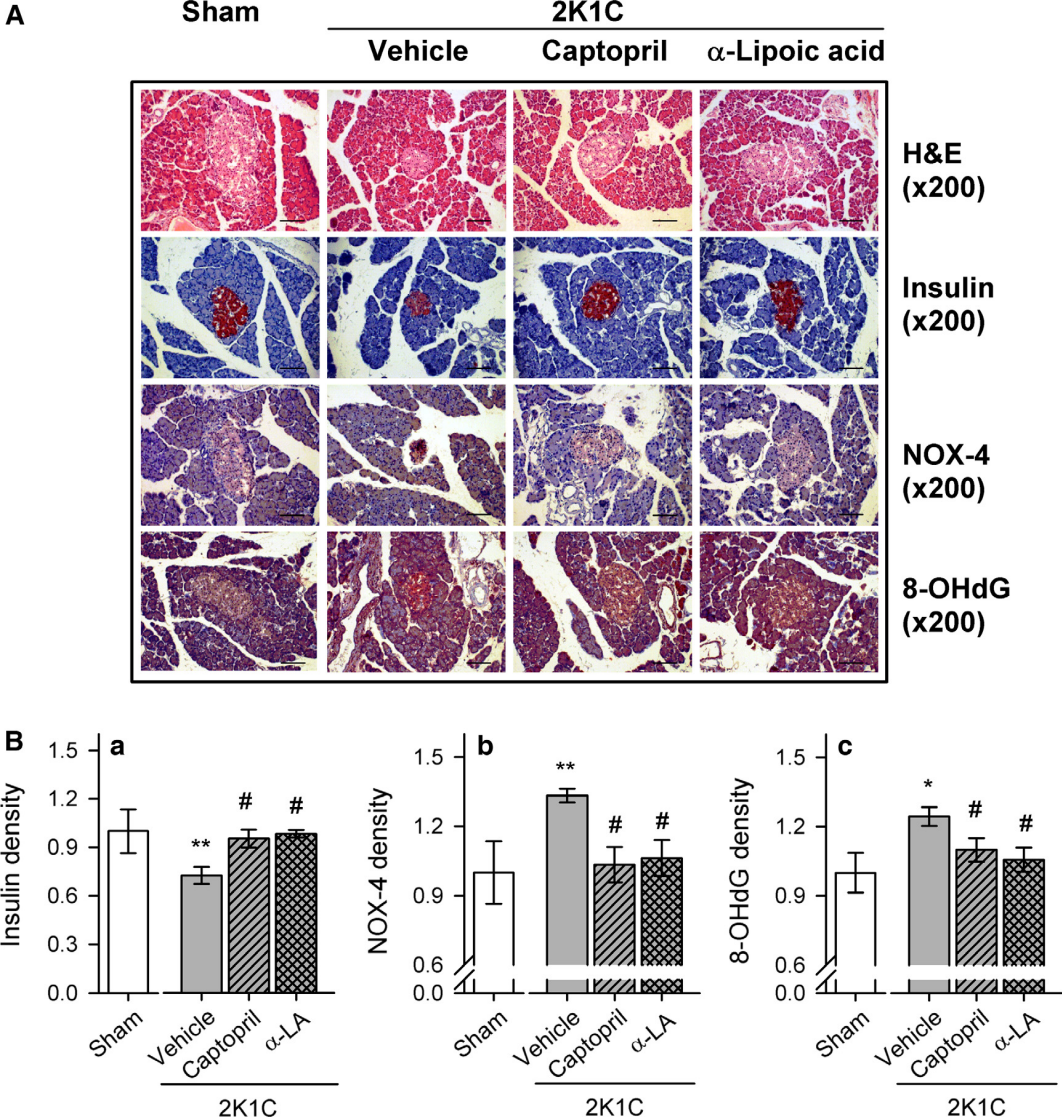
(A) Pancreatic islets immunostained with antibodies against insulin, NOX‐4, or 8‐OHdG in sham and 2K1C rats treated with vehicle, captopril, or *α*‐lipoic acid. (B) Quantification of insulin (a), NOX‐4 (b), and 8‐OHdG (c) staining in pancreatic islets Islet areas and entire section areas were measured using iSolution DT 36 software. Values are expressed as mean ± SEM of five rats in each group. Bar indicates 100 μm. *versus sham group, *P <* *0.05, **P <* *0.01; ^#^versus 2K1C rats fed vehicle, ^#^ *P* < 0.05, ^##^ *P* < 0.01.

Figure 5 shows changes in AT_1_R, AT_2_R, NOX‐4, and SOD expression in the pancreatic tissue of sham and 2K1C rats. AT_1_R (Fig. 5Ba) and NOX‐4 (Fig. 5Bc) expression was increased in the pancreatic tissue of 2K1C rats, and this was reduced by captopril or *α*‐lipoic acid treatment. However, no significant changes in AT_2_R (Fig. 5Bb) and Mn‐SOD (Fig. 5Bd) expression were observed. Pancreatic islets were immunostained with antibodies against the oxidative stress markers NOX‐4 and 8‐OHdG (Fig. 6). NOX‐4 and 8‐OHdG staining was increased in pancreatic islets of 2K1C‐vehicle rats and reduced to control levels in 2K1C‐captopril and 2K1C‐lipoic acid rats.

## Discussion

We have used a hypertension rat model to demonstrate that sustained hypertension induces abnormal islet morphology and insulin secretion in response to high glucose and increases oxidative stress in the pancreas. These effects were reversed by captopril or *α*‐lipoic acid treatment. In addition, we observed a close correlation between blood pressure and oxidative stress in pancreatic tissue. These findings suggest that sustained hypertension increases oxidative stress (ROS generation), thereby altering the structure and function of islets (e.g., *β* cells).

Hypertension is an important underlying symptom of cardiometabolic syndrome (Cersosimo and DeFronzo 2006). Ang II causes insulin resistance by interfering with insulin signaling (Ogihara et al. 2002; Sowers 2004) and impairs insulin sensitivity by suppressing adiponectin production (Ran et al. 2006). 2K1C rats are an established model for early stage Ang II‐dependent hypertension (Morishita et al. 1991). Postop 6‐month 2K1C rats had abnormal OGTT results and insulin response after glucose loading. Moreover, the number and size of islets in pancreas of 2K1C rats and the size of *β*/*α* cells were markedly reduced. High‐glucose‐induced insulin secretion from 2K1C islets was significantly attenuated compared with sham rats. To determine whether hypertension or Ang II is responsible for islet dysfunction, Ang II levels were measured in the plasma. The plasma Ang II level was increased from 43.9 ± 5.51 to 925.5 ± 65.6 pg/mL and 824.2 ± 219.3 pg/mL on postop 5‐week and postop 3‐month 2K1C rats, respectively, and then declined to control level (Table 1). Ang II and aldosterone levels were no different between sham and postop 6‐month 2K1C rats. Morishita et al. (Morishita et al. 1991) reported that in 2K1C rats 4 weeks after clipping, the plasma renin and angiotensin II levels were significantly higher than those in sham‐operated rats. Sixteen weeks after clipping, the plasma renin and angiotensin II levels in 2K1C rats were not significantly higher than those in sham‐operated controls. It has been suggested that the roles of the RAS in maintenance of hypertension in 2K1C rats differ in the acute and chronic phases (Morishita et al. 1991). Therefore, it is possible that the early increase in Ang II levels (i.e., postop 3‐month) could induce substantial damage that was not observed until a later time point. These findings suggest that early increase in Ang II or aldosterone may be partly involved in an impairment of insulin secretion during hypertension and that the duration of hypertension is more important for pancreatic islet dysfunction.

Even though RAS is not activated in essential hypertension, captopril still decreases BP. These data suggest that extrarenal RAS may be more important in the regulation of BP (Morishita et al. 1991; Romero and Reckelhoff 2000; Dinh et al. 2014; Rubattu et al. 2015; Sinha and Dabla 2015). To define whether pancreatic *β*‐cell dysfunction is caused by high blood pressure or oxidative stress, the rats were treated with ACE inhibitor (captopril) or antioxidant (*α*‐lipoic acid). Treatment with captopril or *α*‐lipoic acid improved OGTT results and increased the size and number of pancreatic islets. Treatment also enhanced high‐glucose‐induced insulin secretion. H_2_O_2_ was increased in the pancreas and NOX‐4 expression was higher in pancreatic *β* cells of 2K1C rats; both were decreased by treatment with captopril or *α*‐lipoic acid. Our data are partly consistent with the report showing a role of NOX‐4 in ventricular H_2_O_2_ generation during pulmonary hypertension (Tyagi et al. 2005). Ang II activates NOX‐4, increases production of superoxide, and activates MAPKs in rat pancreatic islets (Alves et al. 2012). However, Ang II levels were not different among groups on postop 6 month. Therefore, Ang II in early stage and/or other factors in late stage of 2K1C rats may induce oxidative stress in the pancreatic *β*‐cell dysfunction. Although blood pressure was not reduced by *α*‐lipoic acid treatment, *α*‐lipoic acid may reduce oxidative stress in the pancreas and restore pancreatic islet function. These results are consistent with previous findings that *α*‐lipoic acid reduces H_2_O_2_‐induced apoptosis in INS‐1 cells and isolated islets (Lee et al. 2009) and improves hyperglycemia through antioxidative properties (Midaoui et al. 2003). Therefore, improving pancreatic *β*‐cell dysfunction may be necessary to reduce oxidative stress and blood pressure in 2K1C rats.

Oxidative stress is a physiological condition and is enhanced by an imbalance between ROS and antioxidants. Therefore, it is important to evaluate ROS levels and antioxidant capacity. AGEs (a source of ROS) and 8‐OHdG (a biomarker of oxidative DNA damage) are associated with age‐related diseases, such as diabetes mellitus (Schleicher et al. 1997) (Dandona et al. 1996) and hypertension (Negishi et al. 1999). In addition to monitor the effects of oxidative stress by the measurement of ROS products, it is also important to evaluate the antioxidant capacity (ORAC). Fruits, vegetables, and low‐fat diet have beneficial effect on BP, serum cholesterol concentrations, and insulin sensitivity by inducing ORAC and suppressing oxidative stress (Botero et al. 2009). A higher ORAC level (antioxidant capacity) is associated with lower risk of hypertension in type 2 diabetes (Rodrigo et al. 2011). In this study, the levels of AGE and 8‐OHdG were substantially increased in the pancreas of 2K1C rats. Moreover, enhanced AGE and 8‐OHdG levels correlated positively with SBP and captopril or *α*‐lipoic acid treatment decreased AGE or 8‐OHdG levels. ORAC was reduced in the pancreas of 2K1C rats and this was reversed by treatment with *α*‐lipoic acid or captopril. Our data are partly consistent with other findings that sustained hypertension stimulates AGE generation and increases 8‐OHdG levels followed by deterioration of pancreatic islet cells, especially *β* cells (Luciano Viviani et al. 2008; Puddu et al. 2010; Gonzalez et al. 2014). Taken together, these results indicate that an imbalance between ROS generation and antioxidant capacity in the pancreas of 2K1C rats causes damage to islet cells.

In conclusion, our findings indicate that sustained hypertension increases pancreatic oxidative stress followed by pancreatic *β*‐cell dysfunction. The relevance of this study in clinical area is that if a person with high blood pressure does not recognize or take antihypertensive drugs during certain period, pancreatic *β* cells may be disrupted and diabetes mellitus will be combined. To prevent the progression of diabetes in hypertension, early treatment of hypertension with antihypertensive drugs and/or antioxidants may be necessary.

## Conflicts of Interest

None declared.
